# The Role of Nicotinamide in Cancer Chemoprevention and Therapy

**DOI:** 10.3390/biom10030477

**Published:** 2020-03-20

**Authors:** Ilias P. Nikas, Stavroula A. Paschou, Han Suk Ryu

**Affiliations:** 1 School of Medicine, European University Cyprus, 2404 Nicosia, Cyprus; i.nikas@euc.ac.cy (I.P.N.); s.a.paschou@gmail.com (S.A.P.); 2 Division of Endocrinology and Diabetes, “Aghia Sophia” Hospital, Medical School, National and Kapodistrian University of Athens, 11527 Athens, Greece; 3 Department of Pathology, Seoul National University Hospital, Seoul 03080, Korea

**Keywords:** niacinamide, NAD^+^ metabolism, SIRT1, PARP1, ONTRAC clinical trial, radiotherapy, non-melanoma skin cancer, actinic keratosis, chemotherapy resistance, cancer prevention

## Abstract

Nicotinamide (NAM) is a water-soluble form of Vitamin B3 (niacin) and a precursor of nicotinamide-adenine dinucleotide (NAD^+^) which regulates cellular energy metabolism. Except for its role in the production of adenosine triphosphate (ATP), NAD^+^ acts as a substrate for several enzymes including sirtuin 1 (SIRT1) and poly ADP-ribose polymerase 1 (PARP1). Notably, NAM is an inhibitor of both SIRT1 and PARP1. Accumulating evidence suggests that NAM plays a role in cancer prevention and therapy. Phase III clinical trials have confirmed its clinical efficacy for non-melanoma skin cancer chemoprevention or as an adjunct to radiotherapy against head and neck, laryngeal, and urinary bladder cancers. Evidence for other cancers has mostly been collected through preclinical research and, in its majority, is not yet evidence-based. NAM has potential as a safe, well-tolerated, and cost-effective agent to be used in cancer chemoprevention and therapy. However, more preclinical studies and clinical trials are needed to fully unravel its value.

## 1. Introduction

Cancer is a multistep process caused by an accumulation of molecular aberrations—activation of oncogenes, inhibition of tumor suppressor genes, epigenetic plasticity—that deregulate intracellular signaling pathways and drive cancer initiation and progression [1,2,3]. At the histologic level, normal tissues give rise to premalignant changes and, when the genetic alterations pile up to a higher number, the latter progress to malignancy (cancer) [1,2,3]. Through this process, cells gradually acquire traits that allow them to sustain uncontrolled proliferation, evade apoptosis, enhance angiogenesis, induce epithelial-mesenchymal transition (EMT), invade, and metastasize [4,5,6,7]. Of them, metastasis is the most common cause of morbidity and mortality [8]. Cancer cells’ ability to reprogram their metabolism is also critical for survival, growth, and metastasis [7]. Cancer metabolism could exhibit deregulated uptake of nutrients, intracellular metabolism, gene expression, and interactions with the microenvironment [9]. To suppress the genetic damage responsible for cancer initiation and progression, cells use diverse DNA repair systems which, if compromised, result in mutations; the latter characterize both premalignant and malignant changes [10]. The immune system is another defense mechanism our body uses against cancer; hence, immunosuppression is associated with higher cancer incidence [11,12,13]. Unfortunately, cancer cells might acquire the ability to evade immune system surveillance [7]. In contrast, chronic inflammation, a component of non-specific immunity, is tumor-promotive rather than suppressive [7,14].

Cancer chemoprevention involves the use of diverse natural, biological or synthetic agents to inhibit cancer initiation or progression [15,16]. Chemopreventive agents could include hormones, medications, vitamins, minerals, and vaccines [15]. Primary chemoprevention refers to the use of such agents in a healthy population, while secondary and tertiary chemoprevention aim to inhibit progression of a premalignant change to cancer or recurrence of an already treated cancer, respectively [16]. For instance, antiestrogens are prescribed after surgical treatment of hormone-positive breast cancer patients to reduce the chance of recurrence [17], and HPV (human papillomavirus) vaccination has been introduced to prevent cervical cancer [18]. Aspirin or other NSAIDs (nonsteroidal anti-inflammatory drugs) have been associated with lower incidence and mortality in colorectal cancer [19]. Metformin, an antidiabetic medication, has recently shown promising results for cancer chemoprevention and therapy [20]. Concerning vitamins, evidence shows that vitamin D could have a preventive role against colorectal cancer [21].

Cancer therapy is traditionally performed with surgery, chemotherapy, and radiotherapy [22]. To select the best possible management, it is important to grade and stage each malignant tumor. Tumor grading refers to its histologic level of aggressiveness [23,24], while staging to its extent into the body (local size and extent, regional lymph node involvement, and distant metastases) [25]. Notably, tumor heterogeneity is also taken into account when treating cancer patients and is the base of cancer precision therapy [26]. This heterogeneity can be found among distinct cancers of the same histologic type, between a primary cancer and its metastasis or among the cellular compartments that compose a tumor mass [27,28]. For instance, hormone-positive cancers are treated differently than HER2 (human epidermal growth factor receptor 2) positive breast cancers or TNBCs (triple-negative breast cancers) [29,30]. Tumor heterogeneity is studied with high throughput molecular tests and targeted therapies are presently standard of care [31,32]. Of interest, as cancer progresses, it accumulates genetic and epigenetic alterations thus becomes more heterogeneous. The latter could be associated with resistance to chemotherapy or targeted therapy [33,34]. Concerning radiotherapy, tumor hypoxia is one of the causes of resistance and agents that enhance tumor blood flow and oxygenation improve therapeutic response [35,36].

This review examines the role of nicotinamide (NAM) in cancer chemoprevention and therapy. It first starts with a brief description of NAM basic principles and metabolism, then continues with the existing evidence on its potential utility in chemoprevention, chemotherapy, and radiotherapy, and finishes with a discussion of the findings in addition to future perspectives.

## 2. NAM Basic Principles and Metabolism

NAM is a water-soluble form of Vitamin B3 (niacin) that can be found in animal and plant food, also as a cost-effective vitamin supplement [37,38,39]. It is absorbed in the stomach or intestine, metabolized in the liver, and excreted through the kidneys [40,41]. While its presence is vital for cellular energy metabolism, lack of NAM is associated with fatigue and anorexia. Pellagra, a disease caused by severe NAM deficiency, is identified by the presence of diarrhea, dementia, and dermatitis and can even lead to death if not treated [37].

**Figure 1:**
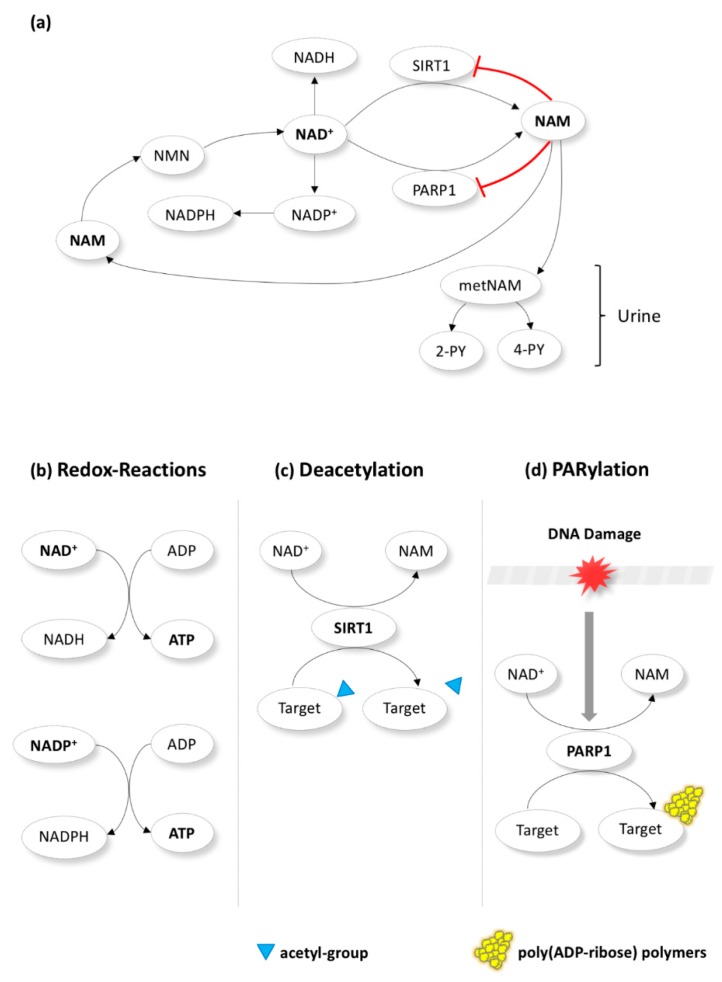
Nicotinamide (NAM) metabolism. NAM is a precursor of nicotinamide-adenine dinucleotide (NAD^+^) and regulates cellular metabolism. NAM is first converted to NMN (nicotinamide mononucleotide) in the cytoplasm before becoming NAD^+^. The latter acts a co-enzyme in redox reactions (**b**) that produce adenosine triphosphate (ATP) or is phosphorylated towards NADP^+^ (which also takes part in redox reactions). Additionally, NAD^+^ is a substrate for enzymes in non-redox reactions, where it is cleaved back to NAM (**a**). The most well-studied enzymes in these reactions are the sirtuin 1 (SIRT1) and the poly ADP-ribose polymerase 1 (PARP1). SIRT1 and PARP1 carry out the protein posttranslational modifications called deacetylation (**c**) and poly(ADP-ribosyl)ation (PARylation)(**d**). Notably, while SIRT1 and PARP1 are activated by NAD^+^ (**c**) and DNA damage (**d**), respectively, NAM suppresses both through a negative feedback mechanism (**a**). NAM produced in non-redox reactions could “go back” to be converted once more to NAD^+^ via NMN. Alternatively, it could be metabolized to metNAM (methyl-NAM) and subsequently to 4-PY (N-methyl-4-pyridone-5-carboxamide) and 2-PY (N-methyl-2-pyridone-5-carboxamide) and excreted through the urine (**a**).

NAM is a precursor of nicotinamide-adenine dinucleotide (NAD^+^), which takes part in several redox and non-redox reactions that regulate cellular energy metabolism (Figure 1) [38,39]. NAD^+^ acts as a co-enzyme in dehydrogenase reactions that result in the production of adenosine triphosphate (ATP). High NAD^+^ (or high NAD^+^/NADH ratio) suppresses the production of reactive oxygen species (ROS) and enhances mitochondrial quality. Therefore, it protects against oxidative stress and improves cell survival [42,43,44]. Except its vital part in redox homeostasis, NAD^+^ is also a substrate for enzymes in non-redox reactions, where it is cleaved back to NAM. The most well-studied enzymes in these reactions are the sirtuin 1 (SIRT1) and the poly ADP-ribose polymerase 1 (PARP1) [38,39]. SIRT1 and PARP1 carry out the protein posttranslational modifications called deacetylation and poly(ADP-ribosyl)ation (PARylation), respectively [38,39]. SIRT1 is a NAD^+^ dependent class III histone deacetylase (HDAC) of histones and various non-histone transcription factors (e.g., p53) involved in diverse intracellular processes including DNA repair, metabolism, apoptosis, proliferation, hormone response, aging, and carcinogenesis [39,45,46]. Whereas NAD^+^ stimulates SIRT1-mediated deacetylation of target proteins, low NAD^+^/NADH ratio diminishes SIRT1 activity [47]. PARP1, triggered by DNA damage (strand breaks), enhances DNA repair and maintains genome stability [40,48]. Of interest, mild and moderate genetic damage facilitate DNA repair or apoptosis, respectively; however, severe damage leads to PARP1 overactivation that depletes NAD^+^ and ATP leading to necrosis [40,47]. Notably, while SIRT1 and PARP1 are activated by NAD^+^ and DNA damage, respectively, NAM suppresses both through a negative feedback mechanism (Figure 1) [38,39].

Several studies have shown that NAM could offer a benefit in several diverse diseases and pathologic conditions [39]. It is reported to have anti-inflammatory properties as it suppresses the expression of several pro-inflammatory mediators or the pro-inflammatory transcription factor NF-κΒ (nuclear factor kappa B) through the latter’s SIRT1-mediated deacetylation [37,39]. NAM is therapeutic for specific inflammatory skin lesions such as bullous pemphigoid and acne vulgaris [49]. In addition, it is regarded as neuroprotectant primarily due to increasing the levels of NAD^+^ [39]. Therefore, NAM could have a therapeutic role against trauma, ischemia, and neurodegeneration—Parkinson, Alzheimer, and Huntington diseases—[50] in addition to neuropsychological disorders including depression and schizophrenia [39,51]. Likewise, NAM could be beneficial against diabetes [52], HIV infection [53], and glaucoma [54]. Concerning cancer, NAM has already shown clinical efficacy against non-melanoma skin cancer (NMSC) [55] and radiotherapy resistance due to tumor hypoxia [35,36].

Oral administration of NAM is safe and well tolerated for doses below 3 g/day [39]. Several clinical trials have shown no significant differences regarding side effects between NAM-treated individuals and the placebo group [40,55,56,57]. In contrast, high oral doses of NAM, especially above 6 g/day, have been associated with gastrointestinal toxicity [40]. Adverse effects such as flushing, hypotension or headache due to vasodilation are believed to be caused by nicotinic acid (another form of vitamin B3, also a NAD^+^ precursor) rather than the NAM itself [39,41,47].

## 3. Nicotinamide and Cancer Chemoprevention

Most relevant studies, at both preclinical and clinical levels, have been performed in the field of skin cancer chemoprevention, especially for non-melanoma skin cancer (NMSC) (Table 1). 

NMSC is the most commonly diagnosed malignancy in Australia/New Zealand and North America, with a global estimated number of 1,042,056 new cases for 2018. Yet, it is characteristically absent from the top ten list of the estimated number of deaths in the same year [81]. Although most NMSCs follow an indolent course, their incidence and associated morbidity is on the rise [82]. NMSC is mainly divided into basal cell carcinoma (BCC) and squamous cell carcinoma (SCC) [83]. Actinic keratosis (AK), a common intra-epidermal lesion found in long-term photo-exposed skin areas, increases someone’s risk to develop NMSC [84,85,86]; around 10% of AKs are estimated to progress to invasive cancer [87]. Ultraviolet (UV) radiation from sunlight is associated with a higher risk of skin cancer [40,47]. The former depletes NAD^+^ thus ATP and cellular energy; hence, UV-induced DNA damage is followed by defective DNA repair and genetic instability [40,47]. Immunosuppression induced by UV radiation is another carcinogenesis factor [41,88,89,90]. This is obvious by the fact that cancer shows higher incidence in transplant recipients [91,92]. Similar to UV radiation, aging also depletes NAD^+^ levels, enhances the formation of ROS and hampers DNA repair; therefore, it promotes genomic instability [47]. As a result, skin cancer affects more commonly the elderly rather than younger patients [93]. 

NAM has exhibited skin cancer chemoprevention characteristics at the preclinical level (cell lines, animal models, human tissues). When administered together with butyric acid and calcium glucarate, it suppressed the DMBA (7,12-dimethylbenz (a) anthracene)-induced formation of skin tumors in animal models [58]. NAM suppressed ATP reduction in UV-irradiated keratinocytes in vitro [59] and enhanced the expression of enzymes involved in cellular energy metabolism [67]. In addition, it stimulated DNA repair when tested in UV-irradiated keratinocytes and ex vivo skin [60,61]; similarly, NAM showed a similar effect on genomic stability when tested in melanocytes [62]. As described before, chronic inflammation is a hallmark of cancer [7]. NAM displayed anti-inflammatory ability by suppressing the expression of diverse pro-inflammatory mediators including interleukins (IL) 1β, 6, 8, and 10, and tumor necrosis factor-alpha (TNF-α) in vitro [63] or by reducing the number of macrophages, which constitute the main regulator of chronic inflammation, in human NMSC tissues of patients treated with NAM [64]. 

NAM has displayed the ability to counteract UV-induced immunosuppression. Gessler et al. showed that topical NAM administration reduced immunosuppression and suppressed tumor formation in UV-irradiated animal models [65]. The same group reported similar results by administering oral niacin instead of topical NAM [94]. Several clinical studies have also confirmed the role of NAM against immunosuppression. Topical or oral NAM administration in human patients reduced immunosuppression in selected UV-irradiated sites, as measured with the Mantoux immunity reaction, compared to the patients that received placebo [66,67,68]. Oral NAM was also safe and well tolerated, while it was absorbed effectively by the tested patients, as shown with the increased NAD^+^ levels in their blood [68]. In addition, Thanos et al. showed that both topical and oral NAM reduced immunosuppression in skin areas undergoing photodynamic therapy [69].

Various clinical studies have exhibited NAM efficacy against skin pre-cancer and cancer. In earlier studies, topical or oral NAM administration reduced the incidence of AK in NAM-treated high-risk individuals compared to the ones that received placebo [70,71]. Notably, most of the information concerning NAM’s role in chemoprevention has derived from the ONTRAC (Oral Nicotinamide To Reduce Actinic Cancer) phase III clinical trial. This was carried out on 386 immunocompetent individuals diagnosed with at least two histologically confirmed NMSCs during the last five years [55]. Participants were divided into two groups, one that was treated with oral NAM and the other one with placebo for 12 months. The study first showed that oral NAM at a dose of 500 mg twice daily is safe and well tolerated. In addition, NAM reduced the incidence of AK and NMSC at significant levels; both BCC and SCC were reduced compared to the placebo group [55]. As part of the ONTRAC trial, oral NAM-treated patients showed no significant effect on their neurocognitive function or life outcome [95]. 

Two recent clinical studies evaluated the efficacy of oral NAM in immunocompromised patients. Although Chen et al. study did not reveal significant results due to its small sample size (*n* = 22), NAM was well tolerated and exhibited a 16% and 35% reduction of AKs and NMSCs, respectively [72]. Drago et al. included immunocompromised patients with kidney or liver transplants and reported that NAM suppressed preexisting AKs and even inhibited the development of new AKs or cancer in these patients [73].

Evidence for NAM chemopreventive role is sparse in cancers other than NMSC and only comes from preclinical studies (Table 1). NAM suppressed the formation of bladder tumors in BBN (N-butyl-N-(4-hydroxybutyl)-nitrosamine)-exposed animal models [74]. It also suppressed lung tumor formation in benzo(a)pyrene-exposed animal models, either when administered alone or synergistically with the glucocorticoid budesonide [75], in addition to urethane-exposed animal models [76]. Likewise, NAM suppressed liver tumor development in thioacetamide-exposed [78] or the formation of pre-neoplastic lesions in DEN (diethylnitrosamine)-exposed animal models, showing it plays a role in the early stages of cancer development [77]. In addition, oral NAM reduced the incidence of non-lymphocytic leukemia in alkylation-exposed animals [79]. Concerning kidney tumorigenesis, NAM treatment has shown both tumor-inhibiting and promoting capacity. Rakieten et al. reported that NAM inhibited the formation of renal tumors in streptozotocin-exposed animal models [80], whereas Rosenberg et al. reported that it enhanced kidney tubular tumor development [96]. Lastly, also in contrast to the previous studies, NAM induced the formation of pancreatic neuroendocrine tumors in animal models when administered together with streptozocin [97]. 

## 4. Nicotinamide and Cancer Therapy

### 4.1. Radiotherapy

Apart from its use in the chemoprevention of NMSCs, high level evidence in the form of randomized clinical trials suggests the clinical efficacy of NAM during radiotherapy of selected cancer types (Table 2). Cancers often show resistance to radiotherapy if they are poorly oxygenated [35]. ARCON (accelerated radiotherapy with carbogen and nicotinamide) could help overcome this issue. Carbogen (98% oxygen, 2% carbon dioxide) and nicotinamide enhance oxygenation and increase blood flow of hypoxic cancers improving the efficacy of radiotherapy [35,36].

Concerning head and neck cancers—a category composed of tumors growing in various sites such as the oral cavity, the oropharynx, and the larynx—ARCON was found to enhance locoregional control in a phase II clinical trial [98]. In addition, ARCON counteracted the negative prognostic impact of anemia in patients with head and neck cancer treated with radiotherapy in a phase III clinical trial. Notably, there was no significant association of hemoglobin levels with locoregional control, overall, and disease-free survival [100]. In contrast, a study by Bernier et al. concluded that ARCON showed no significant therapeutic benefit in terms of local tumor control and tumor response in patients treated with radiotherapy, while it was also accompanied by gastrointestinal toxicity connected with the high doses of NAM (6 g/day) used [99].

ARCON’s clinical efficacy seems more evident in laryngeal and urinary bladder cancers treated with radiotherapy. A phase III clinical trial showed that ARCON improved local tumor control in laryngeal cancer patients, especially in the presence of tumor hypoxia [104,105]. It also enhanced locoregional control and disease-free survival in anemic laryngeal cancer patients in addition to their quality of life after the treatment [106,107]. Likewise, ARCON was found to improve prognosis in patients with highly proliferative laryngeal cancers (detected with Ki-67 immunohistochemistry) [102]. In urinary bladder cancer, Hoskin et al. performed a phase II clinical trial where ARCON was found to be relatively safe and well tolerated; in addition, the latter enhanced local regional control and improved overall survival [108,109]. The same group reported some years later the results of their phase III clinical trial; NAM and carbogen improved overall and disease-free survival at a significant level in patients treated with radiotherapy [110]. 

In contrast to the favorable results in laryngeal and urinary bladder cancers, carbogen and nicotinamide showed no significant therapeutic benefit in terms of overall survival in glioblastoma patients treated with radiotherapy. Furthermore, gastrointestinal toxicity in the form of nausea and vomiting was noticed and linked with the high doses of NAM used in these clinical trials [112,113]. Lastly, ARCON also failed to show any significant therapeutic benefit in terms of tumor response in NSCLC (non-small cell lung cancer) patients in a phase I/II clinical trial [114].

### 4.2. Chemotherapy

In contrast to its role in chemoprevention or as an adjunct to radiotherapy in selected cancers, there is little evidence concerning the clinical efficacy of NAM as a chemotherapeutic regimen. To our knowledge, most of the published studies have been preclinical, whereas only a single clinical trial has been performed (Table 3). 

Most studies were found to involve the breast. An earlier study showed that intraperitoneal NAM suppressed breast cancer volume in animal models [119]. More recently, two research groups reported that NAM suppressed proliferation and enhanced apoptosis when tested in the MC-7 hormone-positive breast cancer cell line; in one of these studies, SIRT1 inactivation by NAM was addressed as the main underlying mechanism [120,121]. Similarly, NAM also inhibited SIRT1 when tested in TNBC cell lines, suppressing cell cycle progression and DNA replication; at the same time, it enhanced apoptosis. Additionally, the same authors reported that NAM suppressed DNA repair by inhibiting PARP1 [122]. In another study, NAM suppressed metastasis to the lungs and brain and prolonged survival of TNBC xenografts through normalizing the NAD^+^/NADH ratio, enhancing autophagy and suppressing AKT/mTORC1 (protein kinase B/mammalian target of rapamycin complex 1) signaling [123]. 

In preclinical melanoma models, NAM was reported to suppress vasculogenic mimicry, a reported poor prognostic factor of melanoma [126]. It also inhibited migration in vitro, also invasion and metastasis in vivo through inhibiting SIRT1, while it improved animals’ survival [127]. Concerning liver-related primary cancers, NAM suppressed proliferation and enhanced apoptosis of HCC (hepatocellular carcinoma) in vitro mainly through stimulating the p53/p21 pathway [77]. Additionally, it reduced HCC growth, serum AFP (A-fetoprotein), and enhanced survival of thioacetamide-exposed animal models via suppressing IGF-1 (insulin-like growth factor 1), a well-known HCC promoter [78]. Similarly, NAM inhibited cell cycle progression, EMT (epithelial-mesenchymal transition), and invasion while it enhanced apoptosis of intrahepatic cholangiocarcinoma (bile duct carcinoma) in vitro [128]. In pancreatic cancer, NAM suppressed proliferation, cell cycle progression, invasion, and enhanced apoptosis in vitro by down-regulating SIRT1, KRAS (Kirsten Rat Sarcoma), and p-AKT (phosphated protein kinase B) [130], while it exhibited an analogous effect when administered in combination with valproate [129]. In urinary bladder cancer, NAM suppressed tumor proliferation, growth, and progression by modulating the expression of Myc—expression of the latter was reduced in cancerous compared to the normal urothelium—and its related genes [74]. When tested in the HeLa cell line, which is a model of cervical cancer, NAM suppressed proliferation and enhanced oxidative stress and apoptosis [132]. Lastly, NAM enhanced the delivery of chemotherapy to colon cancer metastases, when administered together with carbogen, in advanced cancer patients [131].

Notably, NAM seems to be of potential value in selected cancers resistant to chemotherapy. However, all studies have experimented on cell lines and evidence of high value is still absent. Concerning breast cancer, NAM reestablished sensitivity to chemotherapy in resistant hormone-positive [124,125] and TNBC cell lines [125]. NAM-induced suppression of SIRT1 and PARP1 was described as the main mechanism of action [124,125]. NAM also reestablished sensitivity to chemotherapy when tested in resistant pancreatic cancer cell lines [130]. 

Two studies have reported the potential role of NAM in the treatment of hematologic malignancies. Audrito et al. used cell lines derived from patient blood samples showing that NAM inhibited SIRT1, exhibiting an anti-proliferative and pro-apoptotic activity in CLL (chronic lymphocytic leukemia) [133]. Amengual et al. first experimented on preclinical models and found that NAM exhibited a synergistic cytotoxic action against DLBCL (diffuse large B-cell lymphoma) when administered together with a pan I/II deacetylase inhibitor (e.g., vorinostat, romidepsin, or belinostat), although it had a relatively weak effect by itself. The underlying mechanism was the acetylation of BCL6 (B-cell lymphoma 6) and p53, resulting in BCL6 suppression and p53 activation, respectively. The same group subsequently performed a phase I clinical trial, where patients with relapsed lymphoma treated with a combination of NAM and vorinostat. The overall response rate (ORR) was 24%, while about half of the patients demonstrated stable disease (SD) [134].

## 5. Discussion

**Figure 2:**
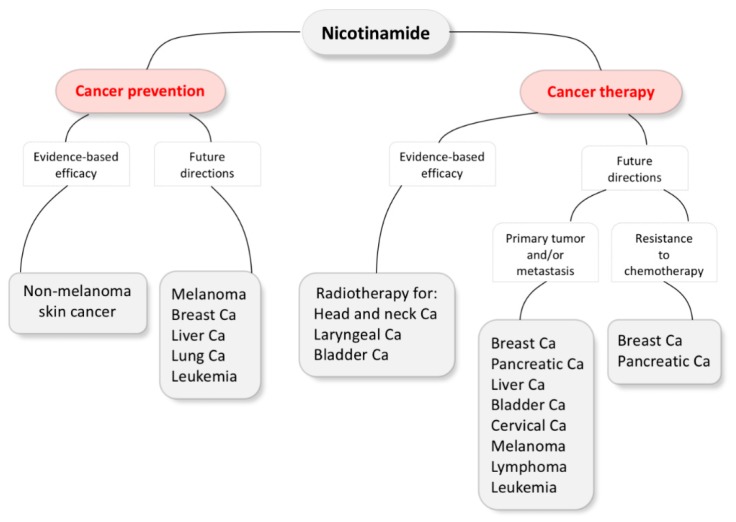
Nicotinamide evidence-based efficacy and future directions in cancer prevention and therapy. Ca, carcinoma.

Accumulating evidence suggests that NAM plays a role in cancer chemoprevention and therapy. Phase III clinical trials have demonstrated the efficacy of NAM in NMSC chemoprevention or as a component of ARCON in the treatment of head and neck, laryngeal and urinary bladder cancers, incorporating their results into clinical practice [55,100,104,105,110]. However, most of the evidence is not yet evidence-based, as it has mostly been acquired in preclinical studies. Nevertheless, it could give future directions with a goal to expand NAM applications in the fields of cancer prevention and therapy (Figure 2).

As shown in the ONTRAC trial, oral NAM is a safe, well-tolerated, and cost-effective chemopreventive agent against both basal and squamous cell skin cancers in high-risk immunocompetent individuals [55]. In addition, it also has value in NMSC prevention in immunocompromised patients with renal or liver transplants [72,73]. Besides the aforementioned studies, a Canadian pilot trial will also evaluate the chemopreventive role of NAM against NMSC in transplant recipients (NCT03769285). 

Thomson et al. showed that NAM enhanced DNA repair in UV-irradiated melanocytes in vitro [62]. However, to our knowledge, no clinical trial has yet assessed the chemopreventive role of NAM against melanoma in high-risk patients. During the ONTRAC trial, a small number of the participants developed melanomas; nevertheless, these were distributed equally between the NAM-treated and placebo groups. In addition, the trial excluded patients with a history of melanoma the last 5 years, as it was designed to study NMSC and its precursor AK. For these reasons, the ONTRAC trial did not draw significant results concerning the role of NAM in melanoma chemoprevention [55,90]. 

NAM could potentially be of value to prevent breast cancer recurrence after primary treatment and a relevant clinical trial could be of value [135]. Likewise, as treated urinary bladder cancers are frequently followed with recurrences [136], a similar clinical trial on such high-risk patients could also end up in significant results. 

NAM’s role in cancer therapy, except for ARCON, has still not been elucidated. A phase I clinical trial, where patients with relapsed lymphoma were treated with a combination of NAM and vorinostat, showed some promising results related to ORR and SD [134]. Of interest, a currently active phase II/III clinical trial is studying the clinical efficacy of NAM when prescribed synergistically with the EGFR (Epidermal growth factor receptor) inhibitors gefitinib or erlotinib in EGFR-mutated advanced NSCLC patients (NCT02416739). 

Notably, NAM could have a role in reestablishing sensitivity to therapy in cases of chemoresistance, as shown in breast and pancreatic preclinical models [124,125,130]. It would also be interesting to investigate if NAM could reestablish treatment sensitivity in cases of resistance to distinct targeted therapies (e.g., tamoxifen or trastuzumab in hormone and HER2 positive breast cancer patients, respectively) or immunotherapy. Lastly, NAM could be of value in the therapy of aggressive cancers that still do not have effective targeted therapy such as TNBCs [122] and more studies in this direction would be desirable.

**Figure 3:**
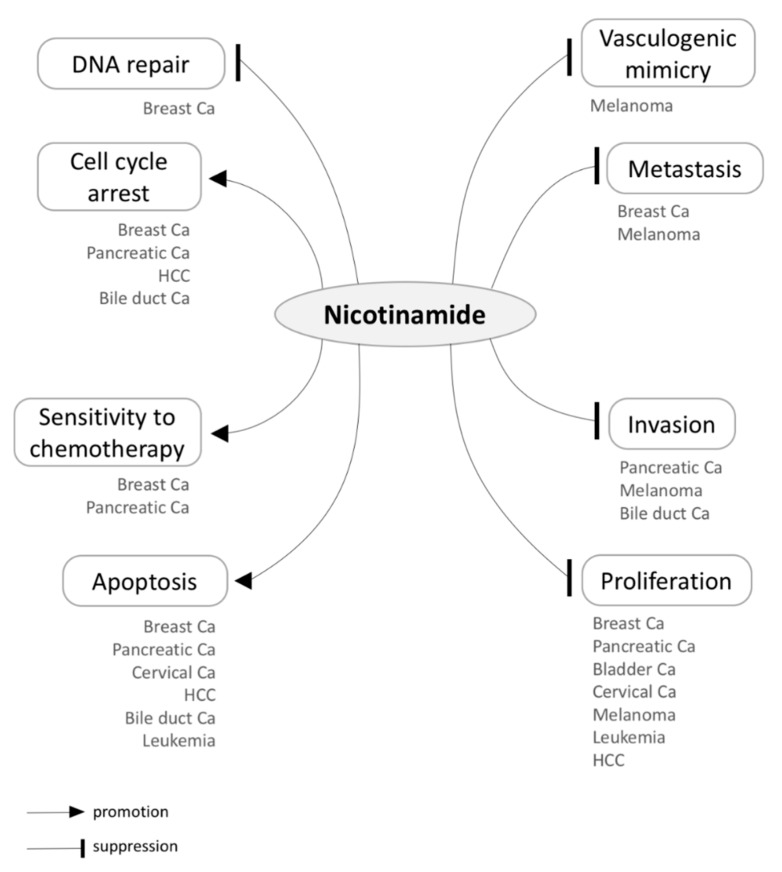
Tumor suppressive effects of Nicotinamide (NAM) in a variety of cancers. Below each of the distinct processes influenced by NAM, the associated cancer types are listed according to the existing literature. HCC, hepatocellular carcinoma; Ca, carcinoma.

Preclinical studies have shown that NAM administration is tumor suppressive, being able to disrupt multiple key processes — e.g., proliferation, apoptosis, invasion, and metastasis — in a variety of cancers (Figure 3 and Table 3). Notably, NAM primarily seems to exhibit its chemotherapeutic capacity by suppressing SIRT1 and PARP1, as already shown in preclinical models of diverse cancers [120,122,124,125,127,130,133]. NAM-induced SIRT1 or PARP1 suppression also enhances sensitivity to chemotherapy in breast cancer cell lines [124,125]. While PARP1 inhibition suppresses DNA repair in cancer cells, SIRT1 inhibition results in p53 activation, suppressing proliferation and inducing cell cycle arrest and apoptosis [122,133,135]. In fact, the NAM-induced activation of the p53/p21 pathway has exhibited antitumor properties in breast cancer, lymphoma, leukemia, and HCC [77,122,133,134]. Besides inhibiting SIRT1 and PARP1, NAM has shown to inhibit the oncogenic KRAS/AKT pathway in skin and pancreatic cancers [58,130], modulate the expression of Myc oncogene in bladder cancer [74], and suppress IGF-1 in HCC [78].

Despite the aforementioned evidence, more studies are needed to unravel NAM’s entire molecular mechanism in diverse cancer scenarios, before its widespread implementation into clinical practice. As previously mentioned, NAM increases the levels of NAD^+^, boosts energy metabolism and protects cells from oxidative stress [39]. NAM also inhibits SIRT1 and PARP1, both of which have been shown to be up-regulated in multiple cancers [39,48,137,138,139]. However, while it suppresses SIRT1, NAM is quickly metabolized into NAD+ which activates SIRT1. Therefore, it is not easy to say if NAM inhibits or in fact stimulates SIRT1 in the long term and more preclinical research in this direction would be vital [38]. To make things more complicated, SIRT1 exhibits a dichotomous behavior and has been described as both tumor promoter and suppressor, while both SIRT1 up-regulation and down-regulation have been associated with cancer progression [45,139,140,141,142]. Some studies have suggested that SIRT1 behavior is dependent on the oncogenic context [142]. For instance, SIRT1 overexpression has been reported to be a poor prognostic factor in TNBCs [143,144] but a favorable one in the luminal A hormone-positive breast cancers [145,146]. In contrast, Tan et al. connected SIRT1 overexpression with shorter survival in luminal cancers [147], while Wu et al. linked it with cancer progression and dismal prognosis in both TNBCs and non-TNBCs [148]. In fact, evidence that supports both a tumor promoter and suppressor function of SIRT1 has been reported for TNBC and luminal breast cancer subtypes [142]. Concerning PARP1, its up-regulation has been reported in multiple cancers, while its inhibition has the capacity to suppress angiogenesis, metastasis, and tumor-induced inflammation [48]. 

Notably, any potential carcinogenic effects in normal tissues induced by NAM administration should be carefully studied. SIRT1 inhibition by NAM could suppress metabolism, a deregulation of which is potentially oncogenic [7,9], while suppression of PARP1 could result in accumulated genetic damage in the long term [39,46]. 

NAM has shown promise as a well-tolerated and cost-effective agent against cancer prevention and therapy. However, more preclinical studies and clinical trials are needed to unravel its clinical value as well as long-term safety.

## Figures and Tables

**Table 1:** Summary of the evidence (preclinical/clinical) that supports the role of nicotinamide (NAM) in cancer chemoprevention.

Tissue/Cancer Type	Level(s) Tested	Summary of Findings	Reference
**Skin**	Animal models	NAM, in synergy with butyric acid and calcium glucarate, suppressed the DMBA-induced tumorigenesis by inhibiting the KRAS/PI3K/AKT signaling pathway and regulating the expression of selected microRNAs	[58]
	Cell lines	NAM suppressed ATP depletion in UV-irradiated keratinocytes	[59]
	Cell lines, ex vivo skin	NAM enhanced DNA repair in UV-irradiated keratinocytes and ex vivo skin	[60]
	Cell lines, ex vivo skin	NAM enhanced DNA repair in sodium arsenite and UV-irradiated keratinocytes and ex vivo skin	[61]
	Cell lines	NAM enhanced DNA repair in UV-irradiated melanocytes	[62]
	Cell lines	NAM suppressed diverse pro-inflammatory mediators in UV-irradiated keratinocytes	[63]
	Human tissues	NAM suppressed the number of macrophages in human NMSC tissues, exhibiting anti-inflammatory capacity	[64]
	Animal models	Topical NAM reduced immunosuppression and suppressed tumor formation in UV-irradiated animal models	[65]
	Patients (clinical study)	Topical NAM reduced immunosuppression in UV-irradiated human skin	[66]
	Patients (clinical study), cell lines	Topical NAM reduced immunosuppression in UV-irradiated human skin; NAM also enhanced energy metabolism and the expression of p53	[67]
	Patients (clinical study)	Oral NAM was well tolerated, while it reduced immunosuppression in UV-irradiated human skin; it also increased NAD^+^ levels in the blood	[68]
	Patients (clinical study)	Topical and oral NAM reduced immunosuppression in skin areas undergoing photodynamic therapy	[69]
	Patients (clinical study)	Topical NAM reduced the incidence of AK	[70]
	Patients (phase II clinical trial)	Oral NAM reduced the incidence of AK	[71]
	Patients (phase III clinical trial)	Oral NAM was safe and well tolerated, while it reduced the incidence of AK, SCC, and BCC in immunocompetent patients	[55]
	Patients (clinical study)	Oral NAM suppressed AKs and cancer in immunocompromised patients	[72]
	Patients (clinical study)	Oral NAM suppressed preexisting AKs in immunocompromised patients, also inhibited the development of new AKs or cancer	[73]
**Urinary bladder**	Animal models, cell lines, human samples (mining of published data)	NAM suppressed bladder tumor formation in BBN-exposed animal models and prevented urinary bladder carcinogenesis by modulating the expression of Myc and its related genes	[74]
**Lung**	Animal models	Dietary NAM suppressed lung tumor formation in benzo(a)pyrene-exposed animal models, either when administered alone or synergistically with budesonide	[75]
	Animal models	Dietary NAM suppressed lung tumor formation in urethane-exposed animal models	[76]
**Liver**	Animal models	NAM inhibited the formation of pre-neoplastic lesions	[77]
	Cell lines, animal models	NAM suppressed liver tumor formation in thioacetamide-exposed animal models	[78]
**Leukemia**	Animal models	Oral NAM reduced the incidence of non-lymphocytic leukemia in alkylation-exposed animal models	[79]
**Kidney**	Animal models	NAM suppressed tumor formation in streptozotocin-exposed animal models	[80]

DMBA, 7, 12-dimethylbenz (a) anthracene; KRAS, Kirsten rat sarcoma; PI3K, phosphatidylinositol-3-kinase; AKT, protein kinase B; ATP, adenosine triphosphate; NMSC, non-melanoma skin cancer; AK, actinic keratosis; SCC, squamous cell carcinoma; BCC, basal cell carcinoma; BBN, N-butyl-N-(4-hydroxybutyl)-nitrosamine.

**Table 2:** Summary of evidence (preclinical/clinical) that supports the role of nicotinamide (NAM) in cancer radiotherapy.

Tissue/Cancer Type	Level(s) Tested	Summary of Findings	Reference(s)
**Head and Neck**	Patients (phase II clinical trial)	ARCON enhanced locoregional tumor control	[98]
	Patients (phase I/II clinical trial)	ARCON showed no significant therapeutic benefit in terms of local tumor control and tumor response; gastrointestinal toxicity was recorded and linked with the high doses of NAM (6 gr/day) used in this trial	[99]
	Patients (phase III clinical trial)	ARCON counteracted the negative prognostic impact of anemia in patients with head and neck squamous cell cancer	[100]
**Larynx**	Animal models	NAM and carbogen reduced tumor hypoxia in animal models treated with radiotherapy	[101]
	Human tissues	ARCON improved prognosis in patients with highly proliferative laryngeal cancers (high Ki-67)	[102]
	Patients (clinical study)	ARCON enhanced local tumor control	[103]
	Patients (phase III clinical trial)	ARCON enhanced local tumor control, especially in the presence of tumor hypoxia	[104,105]
	Patients (phase III clinical trial)	ARCON enhanced locoregional control and disease-free survival in anemic patients with laryngeal carcinoma; it also improved patient quality of life after the radiotherapy treatment	[106,107]
**Urinary Bladder**	Patients (phase II clinical trial)	ARCON was relatively safe and well tolerated; it also enhanced local regional control and improved overall survival	[108,109]
	Patients (phase III clinical trial)	NAM and carbogen improved overall and disease-free survival at a significant level in patients treated with radiotherapy	[110]
**Brain/Glioblastoma**	Patients	NAM and carbogen showed no significant difference in tumor perfusion of glioblastoma patients treated with radiotherapy	[111]
	Patients (phase I/II clinical trial)	NAM and carbogen showed no significant therapeutic benefit in terms of overall survival in glioblastoma patients treated with radiotherapy; gastrointestinal toxicity was recorded and linked with the high doses of NAM used in this trial	[112]
	Patients (phase III clinical trial)	NAM and carbogen showed no significant therapeutic benefit in terms of overall survival in glioblastoma patients treated with radiotherapy; gastrointestinal toxicity was recorded and linked with the high doses of NAM used in this trial	[113]
**Lung/NSCLC**	Patients (phase I/II clinical trial)	ARCON showed no significant therapeutic benefit in terms of tumor response in NSCLC patients	[114]
**Colon/Primary cancer and liver metastasis**	Animal models	NAM and carbogen reduced tumor hypoxia in primary colon cancer and its metastasis in the liver	[115,116]
**Prostate**	Cell lines	NAM reestablished sensitivity to radiotherapy in resistant prostate cancer cell lines	[117]
**Soft tissue/Fibrosarcoma)**	Animal models	NAM and carbogen reduced tumor hypoxia in fibrosarcoma animal models treated with radiotherapy	[118]

ARCON, accelerated radiotherapy with carbogen and nicotinamide; NSCLC, non-small cell lung cancer.

**Table 3:** Summary of evidence (preclinical, clinical) that supports the role of nicotinamide (NAM) in cancer chemotherapy.

Tissue /Cancer Type	Level(s) Tested	Summary of Findings	Reference
**Breast**	Animal models	Intraperitoneal NAM suppressed tumor growth in animal models	[119]
	Cell lines	NAM enhanced apoptosis in hormone-positive breast cancer cells	[120]
	Cell lines	NAM suppressed proliferation and enhanced apoptosis in hormone-positive breast cancer cells	[121]
	Cell lines	NAM suppressed cell cycle progression, DNA repair, and DNA replication, while it enhanced apoptosis of TNBC in vitro	[122]
	Animal models	NAM suppressed metastasis to the lungs and brain and prolonged survival of TNBC animal models	[123]
	Cell lines	NAM reestablished sensitivity to chemotherapy in resistant hormone-positive breast cancer cell lines	[124]
	Cell lines	NAM reestablished sensitivity to chemotherapy in resistant TNBC and hormone-positive breast cancer cell lines	[125]
**Skin/Melanoma**	Cell lines, human tissues	NAM suppressed vasculogenic mimicry and proliferation, but enhanced invasion of melanoma	[126]
	Cell lines, animal models	NAM suppressed migration in vitro, also invasion and metastasis of melanoma in vivo by inhibiting SIRT1	[127]
**Liver/HCC**	Cell lines	NAM suppressed proliferation, while it enhanced apoptosis and cell cycle arrest of HCC in vitro	[77]
	Cell lines, animal models	NAM suppressed HCC growth, reduced serum AFP, and enhanced survival of thioacetamide-exposed animal models	[78]
**Liver/Bile duct carcinoma**	Cell lines	NAM suppressed cell cycle progression, EMT, and invasion, while it enhanced apoptosis of intrahepatic cholangiocarcinoma in vitro	[128]
**Pancreas**	Cell lines	NAM suppressed proliferation and enhanced apoptosis when administered in combination with valproate in vitro	[129]
	Cell lines	NAM suppressed proliferation, cell cycle progression, invasion, and enhanced apoptosis in vitro, while it reestablished sensitivity to chemotherapy in resistant pancreatic cancer cell lines	[130]
**Colon**	Patients (clinical study)	NAM enhanced the delivery of chemotherapy to colon cancer metastases when administered together with carbogen	[131]
**Urinary bladder**	Animal models, cell lines, human samples (mining of published data)	NAM suppressed tumor proliferation, growth, and progression by modulating the expression of Myc and its related genes	[74]
**Cervix**	Cell lines	NAM suppressed proliferation, while it enhanced oxidative stress and apoptosis in vitro	[132]
**Leukemia**	Cell lines (derived from patient blood samples)	NAM suppressed proliferation and enhanced apoptosis in CLL	[133]
**Lymphoma**	Cell lines, animal models, patients (phase I clinical trial)	NAM exhibited a synergistic cytotoxic action against DLBCL when administered together with a pan I/II deacetylase inhibitor (e.g., vorinostat)	[134]

TNBC, triple-negative breast cancer; SIRT1, sirtuin 1; HCC, hepatocellular carcinoma; AFP, A-fetoprotein; EMT, epithelial-mesenchymal transition; CLL, chronic lymphocytic leukemia; DLBCL, diffuse large B-cell lymphoma.
